# Collider Bias Is Only a Partial Explanation for the Obesity Paradox

**DOI:** 10.1097/EDE.0000000000000493

**Published:** 2016-04-13

**Authors:** Matthew Sperrin, Jane Candlish, Ellena Badrick, Andrew Renehan, Iain Buchan

**Affiliations:** From the a Health eResearch Centre, Farr Institute, and b Institute of Cancer Sciences, Manchester Academic Health Science Centre, University of Manchester, Manchester, United Kingdom.

## Abstract

**Background:**

“Obesity paradox” refers to an association between obesity and reduced mortality (contrary to an expected increased mortality). A common explanation is collider stratification bias: unmeasured confounding induced by selection bias. Here, we test this supposition through a realistic generative model.

**Methods:**

We quantify the collider stratification bias in a selected population using counterfactual causal analysis. We illustrate the bias for a range of scenarios, describing associations between exposure (obesity), outcome (mortality), mediator (in this example, diabetes) and an unmeasured confounder.

**Results:**

Collider stratification leads to biased estimation of the causal effect of exposure on outcome. However, the bias is small relative to the causal relationships between the variables.

**Conclusions:**

Collider bias can be a partial explanation of the obesity paradox, but unlikely to be the main explanation for a reverse direction of an association to a true causal relationship. Alternative explanations of the obesity paradox should be explored. See Video Abstract at http://links.lww.com/EDE/B51.

## Abstract 2

Supplemental Digital Content is available in the text.

“Obesity paradox” is the term given to the finding that, in certain populations, people who are obese seem to live longer. This has been observed in patients with coronary artery disease,^1^ heart failure,^2^ and type 2 diabetes,^3,4^ among others.

Proposed explanations for the paradox include^5^: body fat helping patients survive periods of low nutrition; the nonobese population including patients who have lost weight as a result of more severe illness; body mass index (BMI) poorly representing body fat^6^; BMI cut-offs not being appropriate^7^; and obese people being diagnosed earlier.

This article focuses on the collider stratification bias^5,8^ explanation: a correlation induced between an exposure and confounder when stratifying on a third variable (collider) that is associated with, and downstream from, both.^9^ If the confounder also affects the outcome, conditioning on the collider can induce a false, strengthened, or reversed association between exposure and outcome.

Existing literature has demonstrated that collider stratification bias can occur in principle.^5,8^ However, it is not known whether the conditions under which effect reversal occurs are realistic. The aim of this article is to investigate the plausibility of collider stratification bias as an explanation for the obesity paradox under a realistic generative model.

## METHODS

### Derivation

**FIGURE 1.**
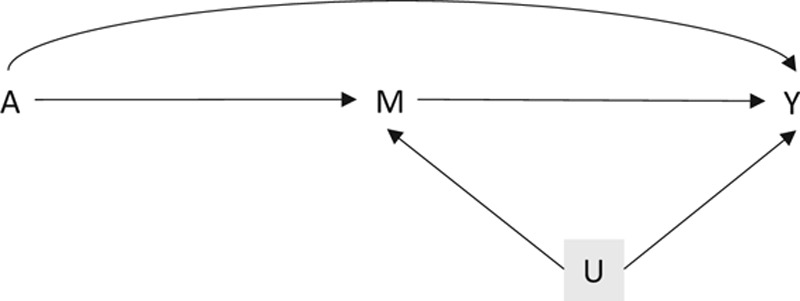
Illustration of collider stratification bias.


We give a general description of collider stratification bias, using the obesity paradox to illustrate, beginning with some definitions from counterfactual causal analysis.^10,11^ Referring to Figure 1, our interest is in the relationship between the exposure *A* (e.g., obesity) and the outcome *Y* (e.g. mortality), complicated by a mediator *M* (e.g., diabetes status) and a confounder *U*, which may be unmeasured. *U* is assumed (unconditionally) independent of *A*. Suppose that *U* and *A* have distributions *F*_*U*_ and *F*_*A*_, respectively. While our derivations allow the variables *U* and *A* to take any form, the mathematics is clearer if we consider the binary case, with 
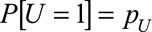
, 
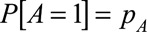
, and the other two variables generated by regression equations:

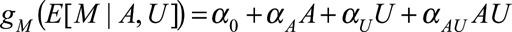
(1)


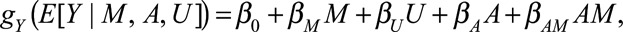
(2)

with 
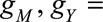
 logit, with inverse: 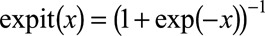.

We are interested in the causal effect of *A* on *Y* (obesity on mortality), conditioned on *M* being at level *m* (diabetes status), i.e., a comparison in which *A* is set (counterfactually) to level *a* or a^*^ (e.g., obese or nonobese):


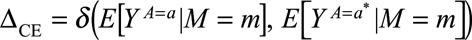


where *δ* represents a difference between the two expectations. However, we calculate the association:


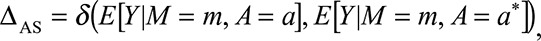


in which we compare individuals *observed* at exposure levels *a* and a^*^. Effect sizes of interest may be a (log) risk ratio or (log) odds ratio. The obesity paradox explained by collider stratification bias argument follows from the noninequality of the above measures. A scenario of particular interest is when the association is the reverse of the causal effect.

The nonequality of *Δ*_CE_ and *Δ*_AS_ is possible because


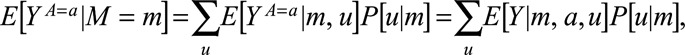


while


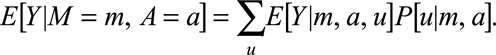


In general, 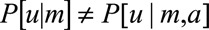 because conditioning on *M* induces a relationship between *U* and *A*.

Following similar lines to the study of Vanderweele,^12^ the above can be computed:


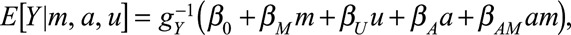



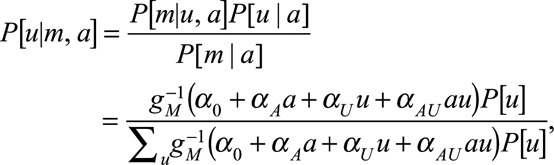



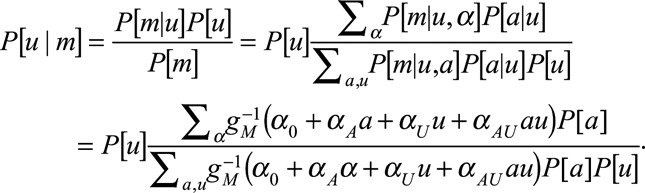


With a logit link, for *Δ*_CE_ we have


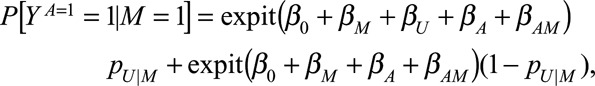


and


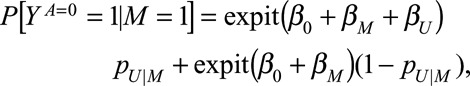


where


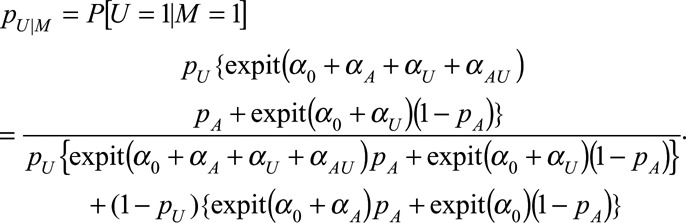


In particular 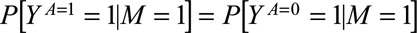 if 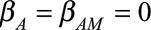.

For *Δ*_AS_:


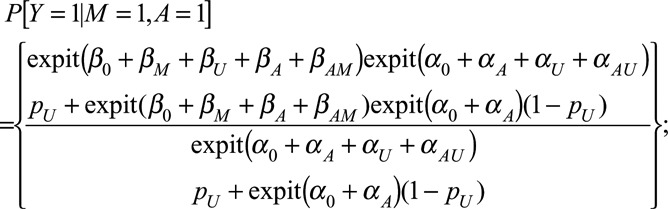


similarly,


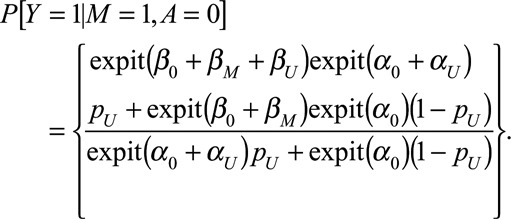


Both the causal effect and association are a weighted average of 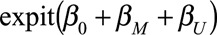 and 
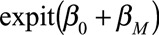
, but the weights differ depending on the status of *A* and *U*, so a spurious association may be observed.

### Illustration

The model is described by Figure 1, and regression Equations (1, 2). We supposed that the only data available are those with *M* = 1 (e.g., those with diabetes). We visualized the discrepancy between the association and causal effect for a range of parameter values. The collider bias variables *α*_*A*_, *α*_*U*_, *α*_*AU*_, and *β*_*U*_ were varied over a grid from −3 to 3; this range captures the salient features, and covers the range of parameters that may reasonably be observed in practice. We considered two scenarios for *β*_*A*_ no causal effect (*β*_*A*_ = 0), and some causal effect (*β*_*A*_ = 1). Throughout we set *p*_*U*_ = *p*_*A*_ = 0.5, 
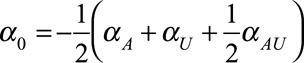
, and 
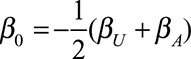
), so that the prevalences of all variables remain close to 50% (e.g., half the population are obese). We set *β*_*M*_ =*β*_*AM*_ = 0, without loss of generality.

We presented all effect sizes as odds ratios. The illustrations were produced using R 3.1.0^13^; code is in eAppendix 1 (http://links.lww.com/EDE/B48).

## RESULTS

**FIGURE 2.**
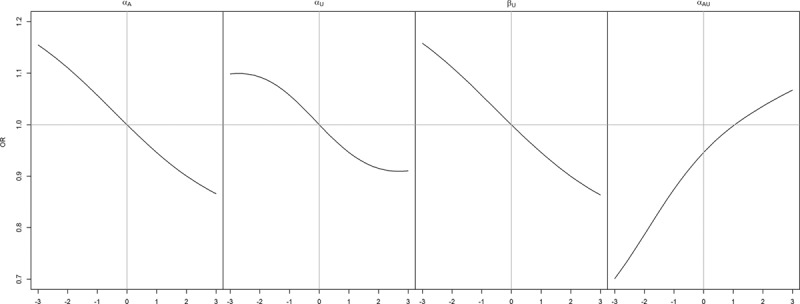
Association (OR) between *A* and *Y* in the null case for varying values of *α*_*A*_,*α*_*U*_, *β*_*U*_, and *α*_*AU*_. In each panel, along the *x* axis, one of these variables is varied from −3 to 3 (*left panel*: *α*_*A*_, *mid-left panel*: *α*_*U*_, *mid-right panel*: *β*_*U*_, *right panel*: *α*_*AU*_), and the other parameters are set to default values.

**FIGURE 3.**
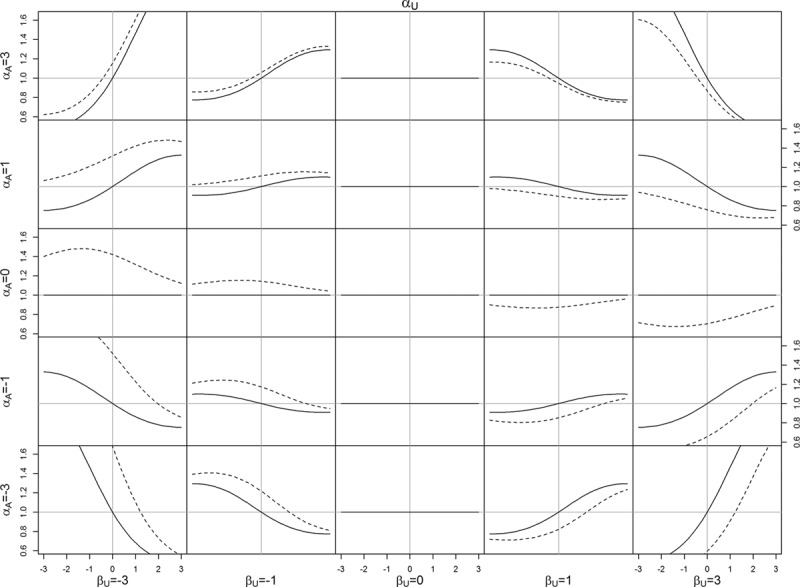
Association (OR) between *A* and *Y* in the null case for varying values of *α*_*A*_,*α*_*U*_, and *β*_*U*_, without interaction (*α*_*AU*_ = 0, *solid line*) and with interaction (*α*_*AU*_ = 1, *dotted line*). Each column (*row*) in the lattice corresponds to the given value of *β*_*U*_ (*α*_*A*_). Within each subgraph, along the *x* axis, *α*_*U*_ is varied from −3 to 3.

Figures 2 and 3 visualize the association between *A* and *Y* when there is no causal effect. Figure 2 looks at the impact of each of the collider stratification bias variables *α*_*A*_, *α*_*U*_, *α*_*AU*_, and *β*_*U*_, one at a time; in each case the remaining variables are set to 1, except *α*_*AU*_ = 0. The left panel of Figure 2 shows that when *α*_*A*_ is positive (with *α*_*U*_ = 1*β*_*U*_ = 1,*α*_*AU*_ = 0), the observed association between *A* and *Y* is negative. This represents a bias because the causal effect is zero. In the diabetes example, this means that in a diabetes population where *A* (obesity) and *U* both increase the risk of *M* (diabetes), and *U* also increases the risk of *Y* (death), but *A* has no effect on *Y* except through *M*, we observe a negative association between *A* and *Y*. Similar results are seen for the other parameters. In Figure 3, each row in the lattice corresponds to a value of *α*_*A*_, while each column corresponds to a value of *β*_*U*_. Within each graph, *α*_*U*_ is varied from −3 to 3, and we consider no interaction (*α*_*AU*_ = 0, solid line) and antagonistic interaction (*α*_*AU*_ = −1, dotted line). The bottom right panels of Figure 3 illustrates that when *α*_*A*_,*α*_*U*_, and *β*_*U*_ parameters are positive, the association between *A* and *Y* becomes negative.

**FIGURE 4.**
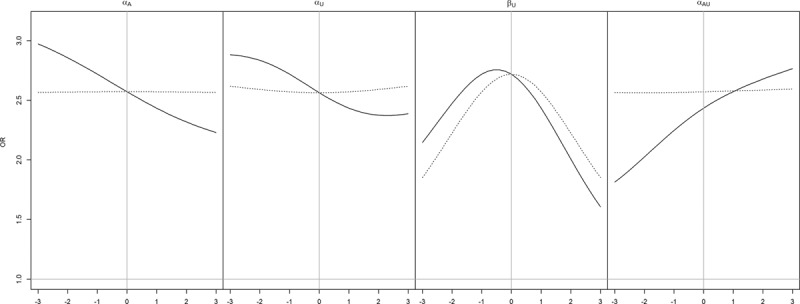
Association (OR) between *A* and *Y* (*solid line*) versus causal effect (log odds) of *A* on *Y* (*dashed line*) for a range of values of *α*_*A*_,*α*_*U*_, *β*_*U*_, and *α*_*AU*_. In each panel, along the *x* axis, one of these variables is varied from −3 to 3 (*left panel*: *α*_*A*_, *mid-left panel*: *α*_*U*_, *mid-right panel*: *β*_*U*_, *right panel*: *α*_*AU*_), and the other parameters are set to default values.

**FIGURE 5.**
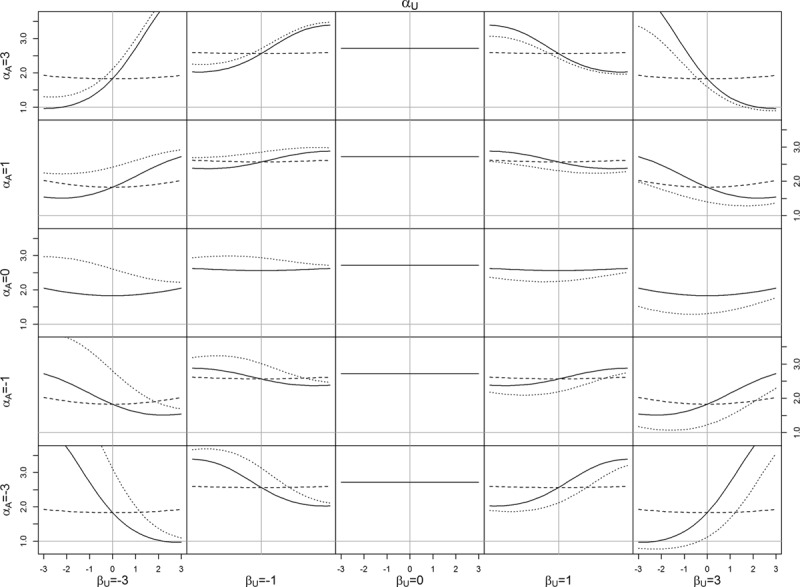
Association (OR) between *A* and *Y* versus causal effect (log odds) of *A* on *Y* for varying values of *α*_*A*_,*α*_*U*_, and *β*_*U*_, with *β*_*B*_ = 1. Each column (*row*) in the lattice corresponds to the given of *β*_*U*_ (*α*_*A*_). Within each subgraph, along the *x* axis, *α*_*U*_ is varied from −3 to 3. Association without interaction (*α*_*AU*_ = 0): *solid line*; association with interaction (*α*_*AU*_ = 1): *dotted line*; causal effect: *dashed line*.

Figures 4 and 5 visualize the association, and causal effect, between *A* and *Y* when there is a causal effect, *β*_*A*_ = 1. Obesity paradox occurs when the association has the opposite sign to the causal effect. The direction of bias between the association and causal effect are the same as when the causal effect was zero. Notably, obesity paradox is happening only for configurations such as *α*_*A*_ = 3, *α*_*U*_ = 3, *β*_*U*_ = 3 (see the bottom right panel of Figure 5), i.e., when all the parameters on the confounding pathway are substantially larger than the causal effect. The association in the reverse direction is small, amplified slightly by antagonism between *A* and *U* (*α*_*AU*_ = −1).

## DISCUSSION

Contrary to much recent literature, our results suggest that collider bias alone cannot fully explain the obesity paradox, with only small discrepancies between the association and the causal effect observed. For large discrepancies to occur (e.g., for the association to reverse the causal effect), the parameters on the collider bias pathway must be large compared with the true causal effect. This could only happen if the true causal effect is small, and therefore unlikely to be important; or the effect of the unmeasured confounder on both the mediator and the outcome is very large, therefore unlikely to be missed from the analysis.

Glymour and Vittinghoff^14^ also demonstrated that collider bias must be very strong to lead to an association that reverses the causal effect, and Greenland^15^ gave a formula for calculating the maximum observable bias. Banack and Kaufman^16^ studied the strength of collider bias required to reverse a particular causal effect. While they concluded that such a reversal was plausible, strong relationships along the collider stratification bias pathway are nevertheless required. Collider stratification bias does not apply when the population is unselected, so our finding is supported by a similar protective effect of obesity in the general population.^17^

For certain nonzero configurations of the model parameters, there is no bias in the estimation of the causal effect (e.g., the crossing of the *x* axis in right panel, Figure 2). This is unfaithfulness, which occurs when a multiplicative model is induced in the risk scale.^14,18,19^

A strength of our study is that our findings are based on mathematical results rather than simulations. However, we restricted to binary variables. Further study is needed to extend this: one context of interest within the obesity paradox is time to event outcome (death), and continuous exposure (BMI).

We have given a simple exposition here, based on a minimal set of four variables. Two of these variables (*U* and *A*) were assumed independent; however, dependence between these variables may affect the degree of the collider bias.^16^ There may be multiple confounding variables; we have considered only one. There may be latent subtypes of the mediating disease.^20^ Finally, we have not considered the time-varying nature of obesity.

When examining the relationship between an exposure and outcome in a subpopulation, the real interest is in whether this relationship differs from the population as a whole, i.e., the relationship is moderated by the mediator. This can only be assessed by modeling the whole population, with interaction terms between exposure and moderator. However, statistical interaction does not necessarily imply a true biological interaction.^21^

Our results show that the paradoxical observation of a protective effect of obesity on mortality is unlikely to be fully explained by collider stratification bias.
